# The expression of protease-activated receptors in esophageal carcinoma cells: the relationship between changes in gene expression and cell proliferation, apoptosis in vitro and growing ability in vivo

**DOI:** 10.1186/s12935-018-0577-0

**Published:** 2018-06-07

**Authors:** Ping Jiang, Shu De Li, Zhi Gang Li, Yue Chun Zhu, Xiao Jia Yi, Si Man Li

**Affiliations:** 1 Department of Pathology, Kunming Medical University, Kunming, 650500 Yunnan China; 2 Department of Biochemistry and Molecular Biology, Kunming Medical University, Kunming, 650500 Yunnan China; 3 Department of Pathology, Second Affiliated Hospital of Kunming Medical University, Kunming, 650000 China

**Keywords:** PAR, CRISPR-CAS9, MTT, Flow cytometry, Nude mice

## Abstract

**Background:**

Protease-activated receptors (PARs) are a family of four G protein-coupled receptors expressed widely in many types of cells. PAR1, 2, and 4 have been shown to play an important role in many of the physiological activities of cells and many types of cancer cells. Esophageal carcinoma has become the fourth most common clinically diagnosed cancer and one of the top three leading causes of cancer-related deaths in China. The functions and expression patterns of PAR1, 2, and 4 in esophageal carcinoma have not published previously.

**Methods:**

Here, we systematically studied the expression of PAR1, 2, and 4 in clinical esophageal carcinoma patients and determined their role in esophageal carcinoma in vivo and in vitro through the overexpression or knockdown of PAR1, 2, and 4.

**Results:**

We found that the expression of PAR1 and 2 expressed higher in esophageal carcinoma than in the paracarcinoma tissues on clinical patients. PAR1 and 2 enhanced cell proliferation both in vivo and in vitro and reduced apoptosis to strengthen cancer cell vitality in TE-1 cells. In contrast, the expression of PAR4 expressed decreased in esophageal carcinoma, and its expression induced apoptosis in vivo and vitro.

**Conclusion:**

In our previous studies and the present study, we noted that the expression of PAR1, 2, and 4 was almost absent in different stages of esophageal carcinoma. PAR1 and 2 might be potential molecular markers for esophageal carcinoma, and PAR4 might be an effective treatment target for esophageal carcinoma prevention and treatment.

## Background

Protease-activated receptors (PARs) are a family of four G protein-coupled receptors that are expressed extensively in many cell types in the human body (e.g., neurons, immune cells, myocytes, platelets, fibroblasts, epithelial cells and endothelial cells). PARs regulate the expression of 2.9% of known human proteins and 1.3% of human genome, and the activation/deactivation of downstream signaling cascades triggered by PARs range from coagulation cascade, inflammation, pain transmission, and repair processes [1]. The presence of PAR1, 2, and 4 promote cell proliferation and migration or apoptosis of types of cancer cells. PAR1 is primarily a thrombin receptor and has been shown to present in human colon cells but not in human colonic epithelial cells breast carcinoma cells, prostate cancer cells, colorectal cancer cells, ovarian cancer cells. Furthermore, the expression of PAR1 is involved in the promotion of tumor cell proliferation and migration. PAR1 and PAR2 both contribute to melanoma cell migration [2]. Also, PAR2 has been proposed to contribute to breast cancer development [3, 4], and cell proliferation and migration in colon cancer [5]. At the same time, PAR4 functions as a suppressor in most tumor cells. The up-regulation of PAR4 induces apoptosis in prostate cancer cells [6], and decreased expression of PAR4 resulted in aggressive gastric cancer [7], breast cancer recurrence and poor prognosis [8, 9], and the promotion of colon cancer cells [10].

Esophageal cancer has become the 4th most common clinical diagnosed cancer and one of the top three leading causes of cancer-related deaths in China [11]. Although the ratio of esophageal cancer only between one-third and one-half in total esophageal cancer, but the overall 5-year survival of esophageal carcinoma ranges from 15 to 25% [12, 13]. The rates of esophageal carcinoma in rural areas were about twice the rate in urban areas. And the low fruit and vegetable intake and unhealthy lifestyle still was the popular cause of esophageal carcinoma genesis. The early prognosis would take a better chance of surviving 5 years after diagnosis. But the prognosis for esophageal carcinoma is poorly dismal in China, and the relative survival rates are about 20% [11]. We hope the research would provide an efficient prognosis target for the esophageal cancer prognosis.

In this study, we aimed to clarify the differences in the expression of PAR1, 2, and 4 between human esophageal epithelial cells and clinical esophageal carcinoma tumor cells. Also, we show the relationships between the regulated expression of PARs (1, 2, and 4) and proliferation and apoptosis in the esophageal carcinoma cell line TE-1. Furthermore, the relationship between the methylation of CpG islands and the expression of PARs need more research. We examined changes of esophageal carcinoma cells growing ability based on the differential expressions of PAR1, 2, and 4 in vivo.

## Materials and methods

### Tissue samples

Tissue samples obtained from 28 cases (male = 21, female = 5, 51–81 years old) the Affiliated Hospitals of Kunming Medical University in Yunnan, China. The diagnosis of esophageal carcinoma based on standard clinical, endoscopic, radiological, and histological criteria. All patients were clean of any chemotherapy or radiation treatment before surgery. The carcinoma tissue samples and corresponding normal control tissue samples (at least 5 cm away from the carcinoma tissue) were quickly obtained during surgery and stored at − 80 °C.

### Cell culture

HEEpiC, TE-1, and TE-10 cells obtained from the Cell Bank of Kunming Institute of Zoology at the China Academy of Sciences. TE-1 and TE-10 cells were cultured in RPMI-1640 (HyClone, Shanghai, China), complete culture medium containing 10% fetal bovine serum (Invitrogen, Shanghai, China) and 1% penicillin–streptomycin and then incubated at 37 °C with 5% CO_2_. HEEpiC cells were cultured with epithelial cell medium 2 (EpiCM2, ScienceCell, China) under similar culture conditions as the TE-1 and TE-10 cells.

### Plasmid construction

To generate stably expressing constructs, the full-length CDS fragments of the PARs (PAR1, 2, and 4) were amplified using the primers shown in Table 1. The Neo-tagged CDS fragments of PAR1, 2, and 4 were subcloned directionally into the *Eco*RI and *Bam*HI sites of the mammalian expression vector pIRES2-EGFP (Invitrogen, Shanghai, China). Also, no-insertion pIRES2-EGFP was used as a vehicle control. After construction, the sequences were confirmed by DNA sequencing.

**Table 1:** Primers for CDS amplification

Gene name	Primer sequences	NCBI accession number
PAR1	F: 5′-GAATTCATGGGGCCGCG-3′	NM_001992.3
	R: 5′-GGATCCCTAAGTTAACAGC-3′	
PAR2	F: 5′-GAATTCATGCGGAGCCCCA-3′	NM_005242.3
	R: 5′-GGATCCTCAATAGGAGGTCTTAA-3′	
PAR4	F: 5′-GAATTCATGTGGGGGCGACT-3′	NM_003950.2
	R: 5′-GGATCCTCACTGGAGCAAAGAGG-3′	

To generate stable high-efficiency PAR interference constructs, the following CRISPR-Cas9 gene knockdown system (Inovogen, Beijing, China) was used to knockdown the PAR1, PAR2, and PAR4 genes. Exon 2 of PAR1, Exon 1 of PAR2, and Exon 2 of PAR4 were selected for the guide RNA design, which was excised using Cas9 with the guide RNA (PAR1: 5′-ACTGTCATGAGCAAGATAG-3′; PAR2: 5′-GGTCATCGTGAACCCCATG-3′; PAR4: 5′-CCTGAGTGCAGTCATGTGG-3′). CRISPR/Cas9 plasmids were constructed according to the manufacturer’s guidelines. Cas9 plasmids with pEGFP-puro were transfected into cells with Lipofectamine 2000 reagents (Life Technologies, Carlsbad, USA).

### TE-1 cell transfection

For pcDNA3.1-PARs and the two vehicle controls, TE-1 cells were incubated in six-well plates at a density of 3.0 × 10^5^ cells/well with the plasmids. After the formation of the complex, the cells were incubated for 12–16 h for Lipofectamine transfection of each plasmid according to the manufacturer’s instructions (Invitrogen, Shanghai, China). The transfection efficiencies were detected visually using an Olympus IX inverted microscope with a fluorescence attachment.

### RNA extraction and polymerase chain reaction (PCR)

RNA extraction and first-strand cDNA synthesis were performed according to previously described methods [14]. Quantitative reverse-transcription PCR (qPCR) was performed using QuantStudio™ 12 K Flex Real-Time PCR System-Time PCR System (Applied Biosystems, Shanghai, China). PCR reactions were carried out with the SYBR Green Real-Time PCR Master Mix (TOYOBO, Shanghai, China) and the reaction conditions were as follows: hold stage at 95 °C for 3 min, 40 cycles of PCR at 95 °C for 2 s and 60 °C for 20 s, and melt curve at 95 °C for 15 s, 60 °C for 1 min and 95 °C for 15 s. The primers used for the amplification of GAPDH, PAR1, PAR2, and PAR4 are shown in Table 2. The relative expression fold change of the mRNAs was calculated using the 2^−ΔΔCt^ method.

**Table 2:** Primers for QPCR

Gene name	Primer sequences	Product (bp)
GAPDH	F: 5′-ATGGGGAAGGTGAAGGTCG-3′	308
	R: 5′-GGGGTCATTGATGGCAACAATA-3′	
PAR1	F: 5′-GCCGCCTGCTTCAGTCTGTGC-3′	648
	R: 5′-GGCCAGACAAGTGAAGGAAGC-3′	
PAR2	F: 5′-CCATCCAAGGAACCAATAGATC-3′	643
	R: 5′-ATGTCTCCCACCAAGAGCTGCTCA-3′	
PAR4	F: 5′-GGCAACCTCTATGGTGCCTA-3′	244
	R: 5′-TTCGACCCAGTACAGCCTTC-3′	

### Western blotting

Proteins of tissue samples and cells were extracted using RIPA solution (Beyotime, Shanghai, China), separated using a resolving gel (12%) and stacking gel (5%) and then transferred onto PVDF membranes (Sigma, Shanghai, China) according to the previously described methods [14]. Antibodies for actin (1:1500 dilution), PAR1 (1:1000 dilution), PAR2 (1:1000 dilution) and PAR4 (1:1000 dilution) obtained from Santa Cruz (Santa Cruz, Shanghai, China). The grayscale analysis was performed using software Quantity One software (Bio-Rad Laboratories).

### MTT assay

To measure cell viability, six TE-1 cells containing PARs expression-regulating plasmids and an equal number of vehicle control cells were equally plated in 96-well plates. Cell viability was determined using the standard MTT dye uptake method. MTT (5 mg/mL) was added and the formazan crystals that formed were dissolved in 10% SDS and 0.01 N HCl. The absorbance was measured at 570 nm with reference to 640 nm using a microplate reader (Infinite^®^ 200 Pro, Tecan, Switzerland) Cell growth was assayed using the MTT Cell Proliferation Kit I (Roche, Shanghai, China) following treatment with the inhibitor for 6, 12, 24, 48 and 72 h.

### Apoptosis assay

Apoptosis was determined with the Cell Meter Annexin V Binding Assay Kit (ATT Bioquest, America) according to the manufacturer’s manual. Briefly, and the cells were treated with gossypol and then 2 × 10^6^ cells were equally harvested. After pretreatment following the manufacturer’s protocol, the TE-1 cells containing PARs expression-regulating plasmids were incubated with 5 μL of Annexin V conjugated with FITC for 10 min at room temperature in the dark. The samples were washed with binding buffer, resuspended in PBS, counter-stained with PI, and analyzed with LSRFortessa (BD, America) and Flowjo 7.6 analysis software.

### In vivo tumor growth and metastasis

Nude mice were purchased from Beijing Vitalriver Laboratory Animal Co. Ltd. and maintained in an SPF laboratory animal room. For the subcutaneous inoculation, different TE-1 cells containing PARs expression-regulating plasmids were resuspended in PBS medium and then 4-week-old nude mice subcutaneously inoculated with 2 × 10^7^ cells. The tumor growth was measured weekly after the appearance of the tumors. The mice were killed 1 month after the inoculation.

### Statistical analysis

All results are presented as the mean ± SD. Comparisons between the different groups were performed using the Student’s two-sample t test. Statistical significance was set at *P *< 0.05. The statistical analysis was performed using SPSS/Win11.0 software (SPSS Inc.).

## Results

### The expression of PAR1 and 2 up-regulated and PAR4 down-regulated in esophageal carcinoma compared to the corresponding normal control tissues

**Fig. 1:**
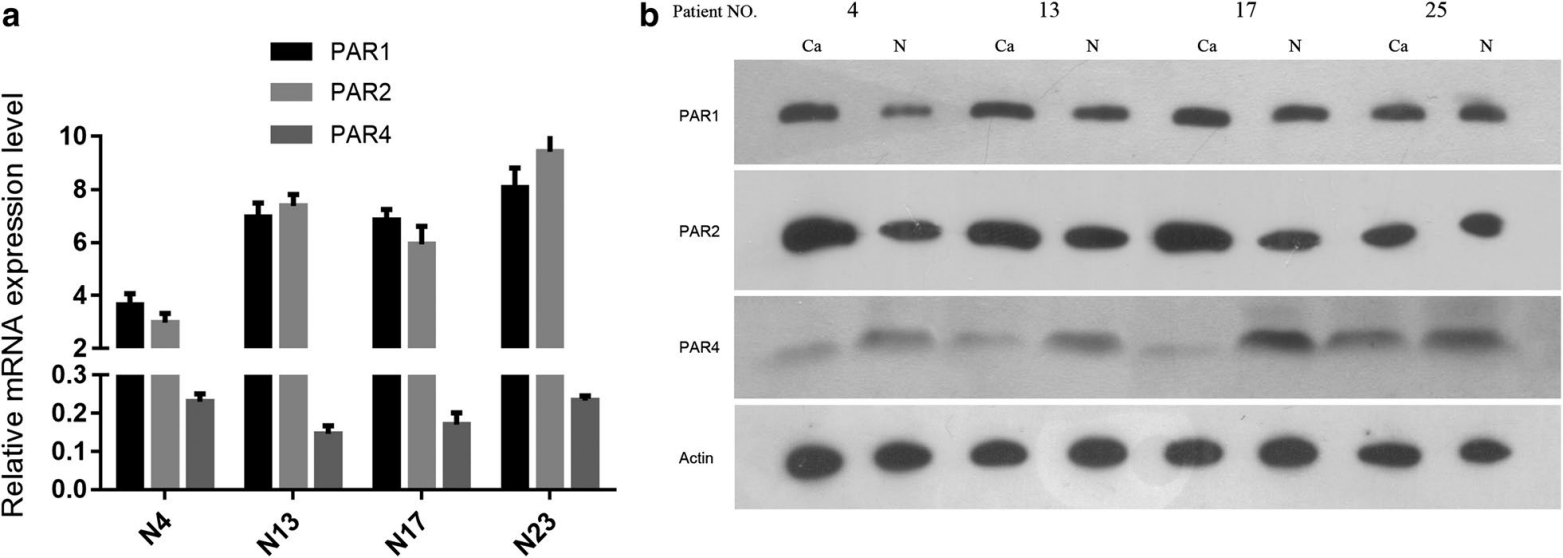
The relative mRNA and protein expression of PARs in ESOPHAGEAL CARCINOMA and control tissues. **a** The mRNA level (relative to GAPDH, n = 3) of PARs in patient cancer tissue compared to each corresponding control tissue (*P *< 0.05); **b** the protein expression of PARs in patient No. 4, 13, 17 and 25 (N = 3, *P *< 0.05)

A total of 28 patients with esophageal cancer participated in this study, and the patient characteristics are summarized in a previous study [14]. Here, we assessed the differences in the expression of PAR1, 2, and 4 between esophageal carcinoma and the corresponding normal tissues of 4 patients randomly sampled from the 28 total patients (Fig. 1). Similar to previous results, the mRNA expression of PAR1 and 2 increased compared to each corresponding control tissue (Fig. 1a). Among the 28 cases of esophageal cancer, 17 (60%) and 20 (71%) cases were found to have increased expression of PAR1 and 2, respectively, compared to each corresponding control tissue. Furthermore, 19 (68%) cases had decreased PAR4 expression. Protein expression was determined using β-actin as the internal reference (Fig. 1b).

Based on the clinical pathological data, the expression of PAR1 was higher in central and lower located esophageal cancer than in upper esophageal cancer (*P *< 0.05). The expression of PAR2 increased in phase III + IV esophageal cancer compared with phase I + II (*P *< 0.05). Further, the expression of PAR4 decreased in lower esophageal cancer compared with central and upper esophageal cancer (*P *= 0.036).

### Overexpression of PAR1, 2 and down-regulation of PAR4 enhanced TE-1 cell viability and reduced apoptosis in vitro

**Fig. 2:**
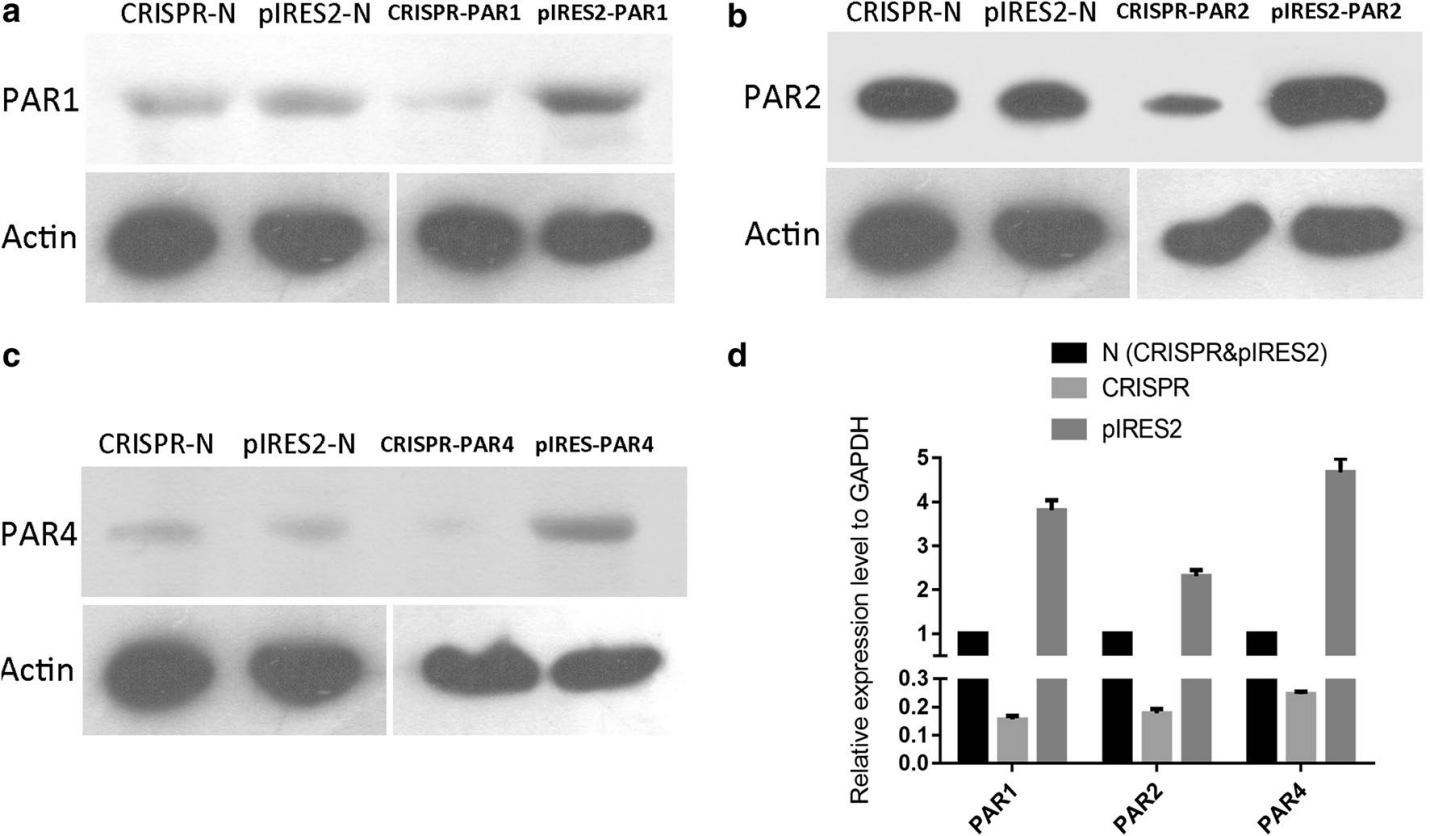
The expression of PAR1, 2, and 4 in different groups of TE-1 cells measured using qPCR and WB. **a**–**c** The protein expression of PARs in TE-1 cells with no-insert CRISPR/Cas9 plasmid vehicle control (CRISPR-N), with no-insert pIRES2 plasmid vehicle control (pIRES2-N), with CRISPR/Cas9 knockdown plasmid (CRISPR-PAR), and with expression plasmid (pIRES2-PAR); **d** the relative mRNA expression of PARs in each group relative to GAPDH in each vehicle control (*P *< 0.05, n = 3)

In the present study, we investigated the role of altered expression of PAR1, 2, and 4 in TE-1 cells using qPCR and western blotting with PAR antibodies. The expression of PAR1, 2 and 4 were significantly up- or down-regulated in the TE-1 cells transfected with expression-regulating plasmids (Fig. 2). As shown in Fig. 2, the mRNA and protein expression of PARs decreased significantly (*P *< 0.05) in the PAR knockdown group (CRISPR group) and increased (*P *< 0.05) in the PAR overexpression group (pIRES2 group).

**Fig. 3:**
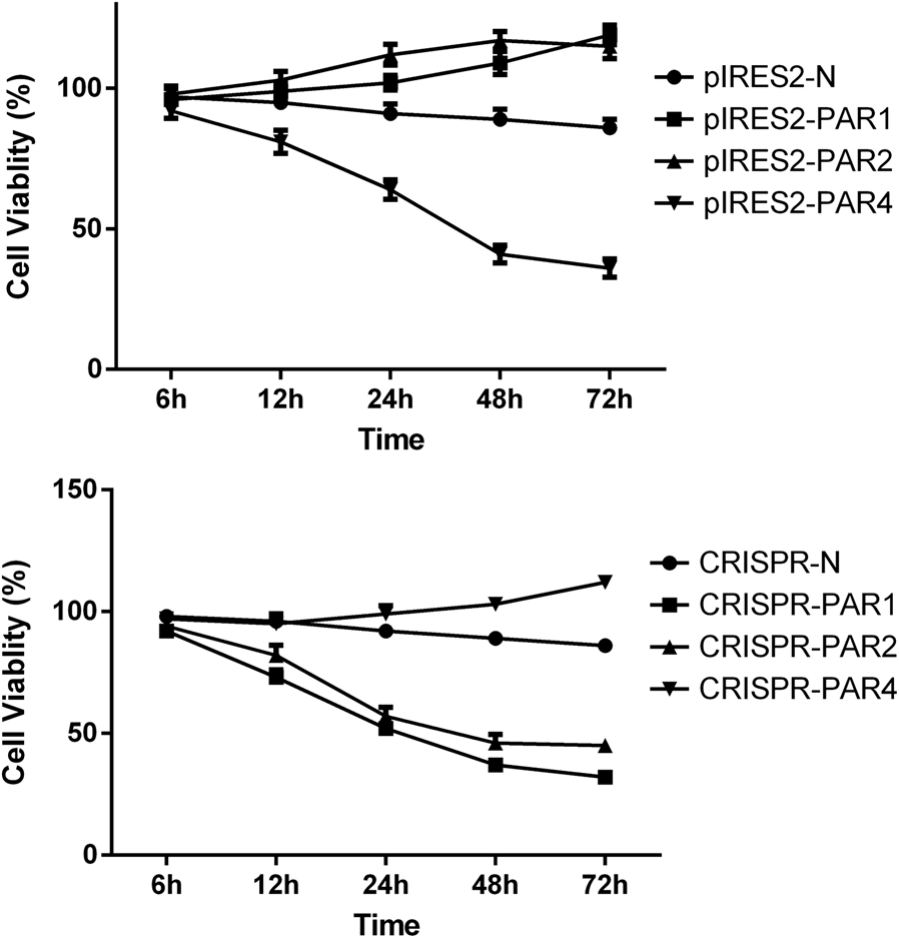
Changes in cell viability in TE-1 cells containing different PAR expression plasmids

To investigate the function of increased or decreased PAR expression in esophageal cancer, we investigated cell growth and apoptosis using MTT and Annexin V assays. As shown in Fig. 3, TE-1 cells with up- or down-regulated PAR expression plasmids were treated for 6, 12, 24, 48 and 72 h and then cell viability was measured using MTT assay. A significant decrease in cell viability was observed in the cells with down-regulated of PAR1 and PAR2 and up-regulated of PAR4 compared to control cells, whereas cell viability increased in the TE-1 cells with up-regulation of PAR1 and PAR2 and down-regulation of PAR4.

TE-1 cells containing different PAR expression plasmids were cultured for 6, 12, 24, 48 and 72 h (n = 3, *P *< 0.05), the data represented the mean ± SE. Cell viability was assessed using the MTT assay and the cell viability value of the vehicle control was set as 100%.

**Fig. 4:**
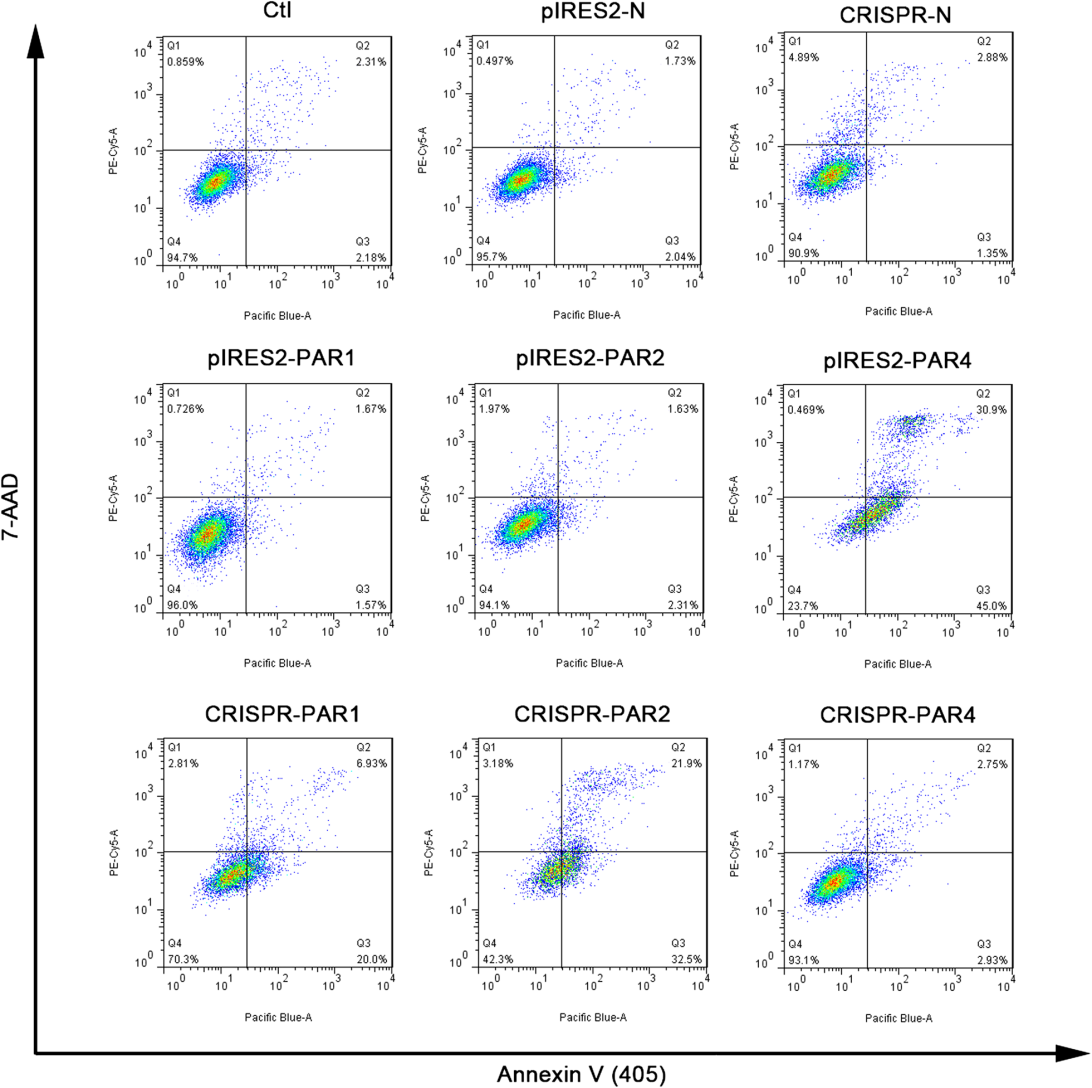
Alterations in apoptosis in TE-1 cells containing different PAR expression plasmids

Double staining with Annexin-V-FITC and 7-AAD was conducted to investigate the induction of apoptosis in the TE-1 cells containing different PAR expression plasmids (Fig. 4). As shown in Fig. 4, there was no difference in early apoptosis in the cells with increased PAR1 and 2 expressions (pIRES2-PAR1: 1.57% and pIRES2-PAR2: 2.32%, respectively) compared to the control cells (2.04%); however, in the cells with overexpression PAR4 (pIRES-PAR4 group), the percentage of early apoptotic cells significantly increased (45–2.04%). In contrast, the PAR1 and 2 knockdown groups (CRISPR-PAR and CRISPR-PAR2, respectively) showed a greater percentage of early apoptotic cells (20.0 and 32.5%, respectively) compared to the CRISPR-N control cells (1.35%). Furthermore, the cells with down-regulated PAR4 expression (CRISPR-PAR4 group) exhibited no significant reduction in apoptosis (2.93%) compared to the CRISPR-N control cells (1.35%).

TE-1 cells containing different PAR expression plasmids were stained with Annexin V and 7-AAD for flow cytometry analysis. The graphs represent the percentage of apoptotic cells (Annexin V and 7-AAD).

### Regulating the expression of PARs influenced esophageal carcinoma growth in vivo

**Fig. 5:**
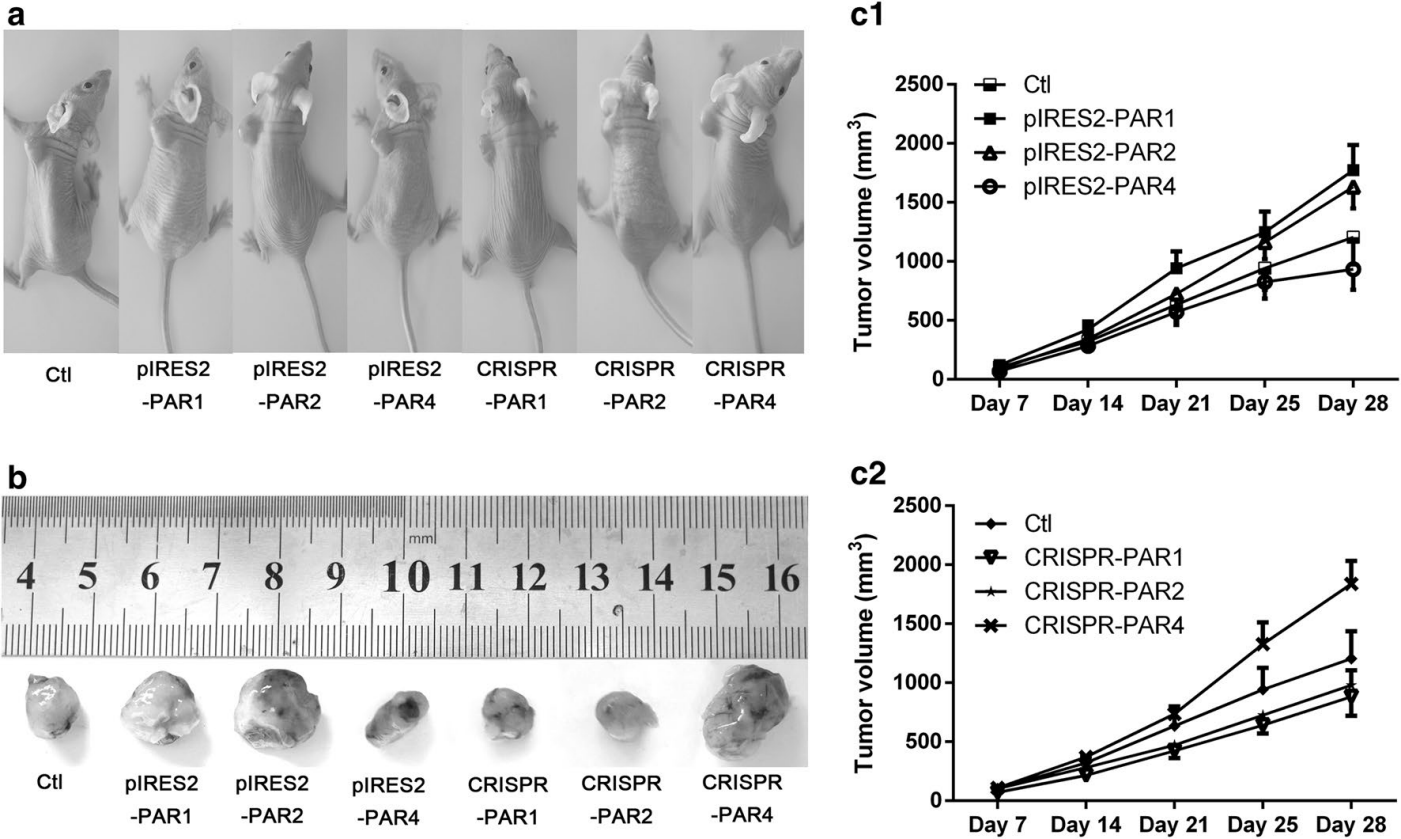
Changes in tumor growth in vivo in TE-1 cells containing different PAR expression plasmids. TE-1 cells containing plasmids for the stable expression or knockdown PARs and control TE-1 cells containing no plasmid were subcutaneously injected into nude mice, and the data are the mean ± SD (n = 5, *P *< 0.05). The mice were sacrificed 4 weeks after the subcutaneous injection. **a** Representative images of tumor development in subcutaneous injected with TE-1 cells. **b** Tumors dissected from the nude mice in each group. c1 and 2, growth curve charts of the tumors from the nude mice in each group

To assess the effect of regulating the expression of PARs on esophageal carcinoma formation in vivo, human esophageal squamous cancer cells (TE-1 cell line, 5 × 10^6^ per mouse) containing PAR1, 2, and 4 expression plasmids were subcutaneously injected into BALB/c nude mice. As shown in Fig. 5, we found that the tumors in the nude mice injected with TE-1 cells containing PAR1 and 2 overexpression plasmids were larger than those in the nude mice injected with control TE-1 cells. The average volume of the tumors in the mice injected with PAR1 overexpression plasmids (group pIRES2-PAR1) was 1774 ± 212 mm^3^ compared with 1206 ± 231 mm^3^ in the mice injected with the control TE-1 cells (n = 5, *P *< 0.05). Similarly, the tumor volume in the nude mice injected with the TE-1 cells containing PAR2 overexpression plasmids was significantly bigger (1632 ± 184 mm^3^) than in the control group. Furthermore, the volume of the tumors in the mice injected with PAR4 overexpression plasmids was smaller (934 ± 176 mm^3^) than in the control group. In contrast, the volumes of tumors in the mice injected with the TE-1 cells containing PAR1, 2, and 4 knockdown plasmids were 883 ± 164, 981 ± 126, 1839 ± 194 mm^3^, respectively. No significant differences were observed among each PAR expression-regulated group.

## Discussion

The present study provides evidence that PAR1, 2, and 4 play an important role in cell proliferation and survival in different types of cancer cells. PAR1 has been confirmed to be widely expressed in human cancers, and studies have been shown that it promotes invasion and tumorigenesis in breast cancer cells [15], mediates ERK1/2 and epidermal growth factor receptor activation to drive cell proliferation in human colon cancer cells [16], and is associated with increased bone metastases in prostate cancer cells [17]. PAR2 is a promiscuous receptor for a broad range of proteases and was also shown to contribute to tumor cell motility and metastasis and protect cancer cells in skin carcinogenesis [2, 18]. PAR4 is a unique pro-apoptotic gene that selectively induces apoptosis in cancer cells [19]. PAR4 shows broad apoptotic functions in prostate cancer cells [20], breast cancer cells, endometrial cancer cells [21], lung cancer cells [22], pancreatic cancer cells [23], and in neuronal cells PAR4 also mediates cell degeneration and pathogenesis [24].

However, the role and expression pattern of PAR1, 2, and 4 in esophageal cancer has not been published. Similar to our previous research, the results of this study indicate that PAR1 and 2 are highly expressed in esophageal cancer compared to the paracarcinoma tissue of clinical patients [20]. Furthermore, in the 28 clinical esophageal carcinoma patients, the expression of PAR1 was higher in central than in upper esophageal cancer and lower in located than in upper esophageal cancer. The expression of PAR2 is higher in late phase esophageal cancer than in early phase. Taken together, the results of this study suggest that increased expression of PAR1 and 2 contribute to the development of esophageal cancer.

In our analysis, the expression of PAR1 and 2 in esophageal cancer increased significantly. PAR1 and 2 in TE-1 cells enhanced cell proliferation and inhibited apoptosis to strengthen cancer cell vitality in vivo. Furthermore, the overexpression of PAR1 and 2 promoted the development of TE-1 cells in nude mice. It is possible that the expressions of PAR1 and 2 are highly associated with more aggressive esophageal carcinoma in nude mice. In contrast, the expression of PAR4 decreased significantly in esophageal cancer compared to the paracarcinoma tissues, and the decreased expression of PAR4 in TE-1 cells enhanced cell growth and decreased the apoptosis both in vivo and in nude mice. These results are in agreement with the role of PAR1, 2, and 4 in other cancers. In our previous work, we found that the expression of PAR4 was associated with tumor differentiation and distant metastasis. Furthermore, the molecular mechanism of altered PAR4 expression is most likely due to the hypermethylation and demethylation of CpG sites in the PAR4 promoter region. However, only a fraction of CpG sites can be methylated or demethylated as part of a coordinated regulatory program in the human genome [25]. The relationship between CpG promoter methylation and the expression of PAR1, 2, and 4 needs further research to discuss.

## Conclusion

The majority of esophageal carcinoma is diagnosed through endoscopy and clinical presentation; however, increasingly essentially asymptomatic cases are being diagnosed [12]. Advanced esophageal carcinoma is generally refractory to radiotherapy or chemotherapy, which leads to poor prognosis [26]. PAR1, 2, and 4 might be potential molecular markers or might be target treatment in clinical treatment for esophageal carcinoma.

## Abbreviation

PAR: protease-activated receptor

## References

[1] Ossovskaya VS, Bunnett NW (2004). Protease-activated receptors: contribution to physiology and disease. Physiol Rev.

[2] Shi X, Gangadharan B, Brass LF, Ruf W, Mueller BM (2004). Protease-activated receptors (PAR1 and PAR2) contribute to tumor cell motility and metastasis11NIH grants CA85405 (BM Mueller), HL16411 (W. Ruf), and HL60742 (W. Ruf). Mol Cancer Res.

[3] Schaffner F, Versteeg HH, Schillert A, Yokota N, Petersen LC, Mueller BM, Ruf W (2010). Cooperation of tissue factor cytoplasmic domain and PAR2 signaling in breast cancer development. Blood.

[4] Parisis N, Metodieva G, Metodiev MV (2013). Pseudopodial and β-arrestin-interacting proteomes from migrating breast cancer cells upon PAR2 activation. J Proteomics.

[5] Hu L, Xia L, Zhou H, Wu B, Mu Y, Wu Y, Yan J (2013). TF/FVIIa/PAR2 promotes cell proliferation and migration via PKCα and ERK-dependent c-Jun/AP-1 pathway in colon cancer cell line SW620. Tumor Biol.

[6] Srinivasan S, Ranga RS, Burikhanov R, Han S-S, Chendil D (2007). Par-4-dependent apoptosis by the dietary compound withaferin A in prostate cancer cells. Can Res.

[7] Zhang Y, Yu G, Jiang P, Xiang Y, Li W, Lee W, Zhang Y (2011). Decreased expression of protease-activated receptor 4 in human gastric cancer. Int J Biochem Cell Biol.

[8] Alvarez JV, Pan TC, Ruth J, Feng Y, Zhou A, Pant D, Grimley JS, Wandless TJ, DeMichele A, Chodosh LA (2013). Par-4 downregulation promotes breast cancer recurrence by preventing multinucleation following targeted therapy. Cancer Cell.

[9] Nagai MA, Gerhard R, Salaorni S, Fregnani J, Nonogaki S, Netto MM, Soares FA (2010). Down-regulation of the candidate tumor suppressor gene PAR-4 is associated with poor prognosis in breast cancer. Int J Oncol.

[10] Gratio V, Walker F, Lehy T, Laburthe M, Darmoul D (2009). Aberrant expression of proteinase-activated receptor 4 promotes colon cancer cell proliferation through a persistent signaling that involves Src and ErbB-2 kinase. Int J Cancer.

[11] Chen W, Zheng R, Baade PD, Zhang S, Zeng H, Bray F, Jemal A, Yu XQ, He J (2016). Cancer statistics in China, 2015. CA Cancer J Clin.

[12] Rustgi AK, El-Serag HB (2014). Esophageal carcinoma. N Engl J Med.

[13] Pennathur A, Gibson MK, Jobe BA, Luketich JD (2013). Oesophageal carcinoma. Lancet.

[14] Li SM, Jiang P, Xiang Y, Wang WW, Zhu YC, Feng WY, Li SD, Yu GY (2014). Protease-activated receptor (PAR) 1, PAR2 and PAR4 expressions in esophageal squamous cell carcinoma. Zoolog Res.

[15] Boire A, Covic L, Agarwal A, Jacques S, Sherifi S, Kuliopulos A (2005). PAR1 is a matrix metalloprotease-1 receptor that promotes invasion and tumorigenesis of breast cancer cells. Cell.

[16] Darmoul D, Gratio V, Devaud H, Peiretti F, Laburthe M (2004). Activation of proteinase-activated receptor 1 promotes human colon cancer cell proliferation through epidermal growth factor receptor transactivation. Mol Cancer Res.

[17] Chay CH, Cooper CR, Gendernalik JD, Dhanasekaran SM, Chinnaiyan AM, Rubin MA, Schmaier AH, Pienta KJ (2002). A functional thrombin receptor (PAR1) is expressed on bone-derived prostate cancer cell lines. Urology.

[18] Rattenholl A, Seeliger S, Buddenkotte J, Schön M, Schön MP, Ständer S, Vergnolle N, Steinhoff M (2007). Proteinase-activated receptor-2 (PAR2): a tumor suppressor in skin carcinogenesis. J Invest Dermatol.

[19] Ranganathan P, Rangnekar VM (2005). Regulation of cancer cell survival by Par-4. Ann N Y Acad Sci.

[20] Chakraborty M, Qiu SG, Vasudevan KM, Rangnekar VM (2001). Par-4 drives trafficking and activation of Fas and Fasl to induce prostate cancer cell apoptosis and tumor regression. Can Res.

[21] Moreno-Bueno G, Fernandez-Marcos PJ, Collado M, Tendero MJ, Rodriguez-Pinilla SM, Garcia-Cao I, Hardisson D, Diaz-Meco MT, Moscat J, Serrano M (2007). Inactivation of the candidate tumor suppressor par-4 in endometrial cancer. Can Res.

[22] Joshi J, Fernandez-Marcos PJ, Galvez A, Amanchy R, Linares JF, Duran A, Pathrose P, Leitges M, Cañamero M, Collado M (2008). Par-4 inhibits Akt and suppresses Ras-induced lung tumorigenesis. EMBO J.

[23] Azmi AS, Aboukameel A, Bao B, Sarkar FH, Philip PA, Kauffman M, Shacham S, Mohammad RM (2013). Selective inhibitors of nuclear export block pancreatic cancer cell proliferation and reduce tumor growth in mice. Gastroenterology.

[24] Guo Q, Fu W, Xie J, Luo H, Sells SF, Geddes J, Bondada V, Rangnekar VM, Mattson MP (1998). Par-4 is a mediator of neuronal degeneration associated with the pathogenesis of Alzheimer disease. Nat Med.

[25] Ziller MJ, Gu H, Müller F, Donaghey J, Tsai LTY, Kohlbacher O, De Jager PL, Rosen ED, Bennett DA, Bernstein BE (2013). Charting a dynamic DNA methylation landscape of the human genome. Nature.

[26] van Hagen P, Hulshof M, Van Lanschot J, Steyerberg E, Henegouwen MVB, Wijnhoven B, Richel D, Nieuwenhuijzen G, Hospers G, Bonenkamp J (2012). Preoperative chemoradiotherapy for esophageal or junctional cancer. N Engl J Med.

